# Evaluation of an interphalangeal-joint prosthetic hand in trans-radial prosthesis users

**DOI:** 10.1080/07853890.2023.2166979

**Published:** 2023-01-16

**Authors:** Natthawan Sutthison, Kazuhiko Sasaki, Gary Guerra, Sirarat Chaisumritchoke, Wisavaporn Niamsang, Thanatat Charatrungolan

**Affiliations:** a Sirindhorn School of Prosthetics and Orthotics, Faculty of Medicine, Siriraj Hospital, Mahidol University, Bangkok, Thailand; b Prosthetic and Orthotic Unit, Sirindhorn National Medical Rehabilitation Institute, Nonthaburi, Thailand; c Department of Exercise and Sport Science, St. Mary’s University, San Antonio, TX, USA

**Keywords:** Trans-radial, upper limb prosthesis, nine hole peg test, QUEST, BAM-ULA

## Abstract

**Background:**

A body-powered functional and cosmetically appeasing hand with moving interphalangeal joints (IPJ-Hand) was created. Function and satisfaction with the IPJ-Hand, conventional hand (CH) and functional hook (FH) in trans-radial prosthesis users were evaluated.

**Methods:**

Eight participants with trans-radial amputations were provided with three prosthetic hands and performed the Nine Hole Peg Test (NHPT), Brief Activity Measure of Upper Limb Amputees (BAM-ULA) and Quebec User Evaluation of Satisfaction with Assistive Technology QUEST).

**Results:**

The data are shown as the median and interquartile range (IQR) due to skewed data distribution. Differences across hands were seen in NHPT with CH 57 (13.3), FH 49.5 (26.5), IPJ-H and 49 (14.8) seconds respectively. BAM-ULA, 10 (1.5), FH 10 (0.7), and IPJ-Hand 10 (1.7). QUEST scores, FH 28.5 (7.2) with the highest score, IPJ-Hand 26 (6.8), and lastly CH 25.5 (9.2).

**Conclusion:**

Individual participant results varied, with some participants performing better on the NHPT when using the IPJ-Hand when compared to the CH and FH. No differences between hands on the BAM-ULA were seen, and although no large differences in QUEST were observed, users performed best using IPJ-Hand.

**Key messages**
- An interphalangeal joint prosthetic hand (IPJ-Hand) offers the similar function of a prosthetic hook, and the appearance of a conventional hand, but with improved dexterity.
- Minimal resources are needed to create the IPJ-Hand for prosthesis wearers.

## Introduction

Upper limb amputation accounts for approximately 8% of all major limb amputations [1] and is typically a result of trauma [2]. Although upper limb amputations make up a small percentage of all amputations, by the year 2050 an estimated 3.6 million individuals will be living with amputation [1]. Upper limb amputation has been reported to be as high as 68.6% of trauma-related injuries [3]. In fact, the incidence of upper limb amputations has seen a decline as a result of limb salvaging surgeries [4] and possibly a reduction in occupational safety [5].

Fortunately, a number of prosthetic treatments are available for individuals with upper limb amputation. Prosthetic restoration is accomplished through body-powered or externally powered devices. Body-powered devices employ the use of a cabling and strap system tethered to the device which opens and closes the hand or hook *via* user body movements [6]. In contrast, externally powered devices require motors and batteries relying on either surface electrodes sensing muscle contractions, electroencephalogram (EEG) or brain-computer interface BCI technologies [7–10].

Both types of prosthesis, body or externally powered, can offer both function and cosmesis. Yet because externally powered devices typically leverage the use of myoelectric hands with interphalangeal joints and cosmetic gloves, they generally have distinct advantages over body-powered devices [11]. This is because for years body-powered terminal device options have been limited to either the functional hook that is well suited for large dexterous tasks or the functional hook that permits similar function with great aesthetics but limited fine motor dexterity. Recently, great emphasis has been placed on the development of better upper limb prostheses as users have had high rates of prosthesis rejection [12,13], and have desired affordable life-like hands [14]. In fact, rejection can be exacerbated for those with higher-level amputations exerting greater effort for device use [15]. There are a number of new externally powered myoelectric hands which can provide both function and cosmesis, but these are cost-prohibitive for the majority of upper limb amputees in the world. Users residing in resource-limited environments (RLE) must choose between a functional hook that offers tactility or a functional hook that provides cosmesis but with reduced function.

Although upper limb amputations generally make up a small percentage of the amputee population [16], several products, such as Otto Bock’s Michelangelo arm have been developed with computerized interphalangeal prosthetic joints (IPJ) and a cosmetic glove cover [17]. These technologies have met user needs for enhanced tactility and cosmesis but at an out-of-pocket price point of approximately $40–60,000 USD. However, is the additional cost worthwhile in terms of user function and satisfaction? Several studies have looked at both of these important facets. A recent study evaluated the US Department of Defense’s advanced prosthesis rehabilitation and technology program. When comparing amputee veterans of the Vietnam conflict to those from recent conflicts, a shift from the use of body-powered to externally powered devices was observed [18]. Another study found users exhibiting great satisfaction with powered multi-function myoelectric upper limb prosthesis [19].

Further evidence to support the importance of marrying both function and cosmesis is evident in a recent study exploring the long-term effects of the Michelangelo hand on functional and psychosocial measures. These authors found consistently high scores in the Box and Block Test (BBT) and Trinity Amputation and Prosthesis Experience Scales (TAPES) [17]. However, a recent review of myoelectric and body-powered prosthesis by Carey et al. noted insufficient evidence to support one type of device over another, emphasizing device selection be based on a patients unique needs [7].

Still, it is clear that a body-powered terminal device providing both dexterity as well as cosmesis might serve a purpose for users residing in RLE. As such, we constructed a body-powered functional and cosmetically appeasing hand with moving interphalangeal joints (IPJ-Hand). Our objective was to evaluate function and satisfaction with the IPJ-Hand, conventional hand and functional hook in a sample of trans-radial prosthesis users.

## Methods

### Participants

This study was approved by the Siriraj Hospital Faculty of Medicine IRB (COA no. SI 902/2020), and all participants provided informed consent prior to study participation. Eligible participants needed to have either a unilateral or bilateral trans-radial amputation, experience using a prosthesis ≥ 1 year, no physiological or musculoskeletal issues, and normal limb ranges of motion. A convenient sample of eight participants, six male and two female traumatic trans-radial amputees 49.87 ± 12.72 years of age were recruited for this study (Table 1).

**Table 1.** Participant demographics.

Participant	1	2	3	4	5	6	7	8
Gender	Male	Male	Male	Female	Female	Male	Male	Male
Age (years)	52	56	39	50	61	69	29	43
Amputation	Right	Right	Right	Right	Right	Left	Right	Right
Socket	NW	Conventional	NW	Munster	NW	NW	Conventional	NW
Previous TD	None	Hook	Myoelectric	Passive	Hand	Hook	Functional hook	Hook
Harnessing	Figure 9	Figure 8	Figure 9	Figure 9	Figure 9	Figure 8	Figure 8	Figure 8

Note: Amputation: amputation side; TD: terminal device; NW: Northwestern; Myo: myoelectric.

### IPJ-Hand

**Figure 1.**
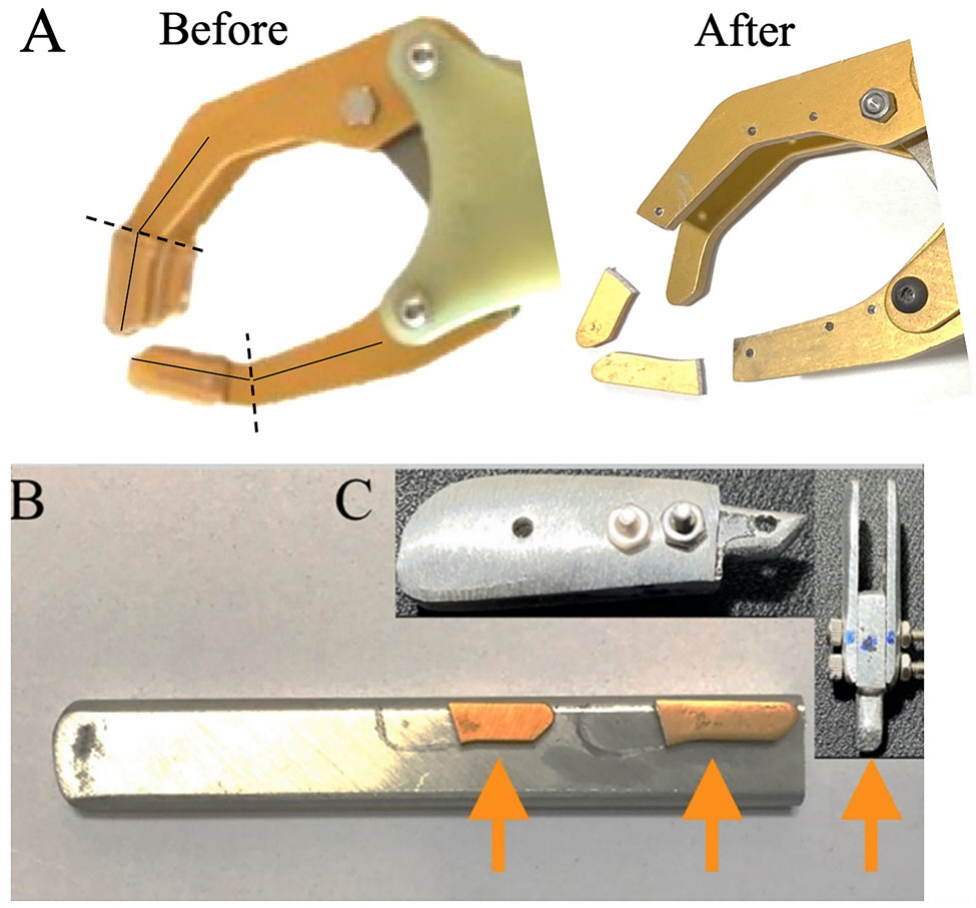
Images illustrating completed steps of the fabrication process for the IPJ-Hand. A voluntary opening prosthetic hand is sourced and fabrication begins by cutting off at the estimated mechanical interphalangeal joint locations of approximately 1.27 cm of the index and thumb fingers (Figure 1(A)). These pieces are not to be discarded as they will now serve as a template for creating six aluminum alloy extension pieces (Figure 1(B)). The exact contours of these extensions should follow that of the hands remaining fingers. This step is part art and requires only that the dimensions of each piece be a length of approximately 21 mm, a width of 10 mm and a height of 1.5 mm. Next, a series of 0.3 mm holes must be punched into the extension bars which serve as a means for attachment back onto the hand. Three bolts along with nuts are required so that the extensions can be pieced together back onto the hand. The dimension of the bolt was chosen so as to permit the hook of the springs to easily catch onto the bolts (Figure 1(C)). Orange arrows designate pieces which need to be cut and used to assemble the interphalangeal joint.

**Figure 2.**
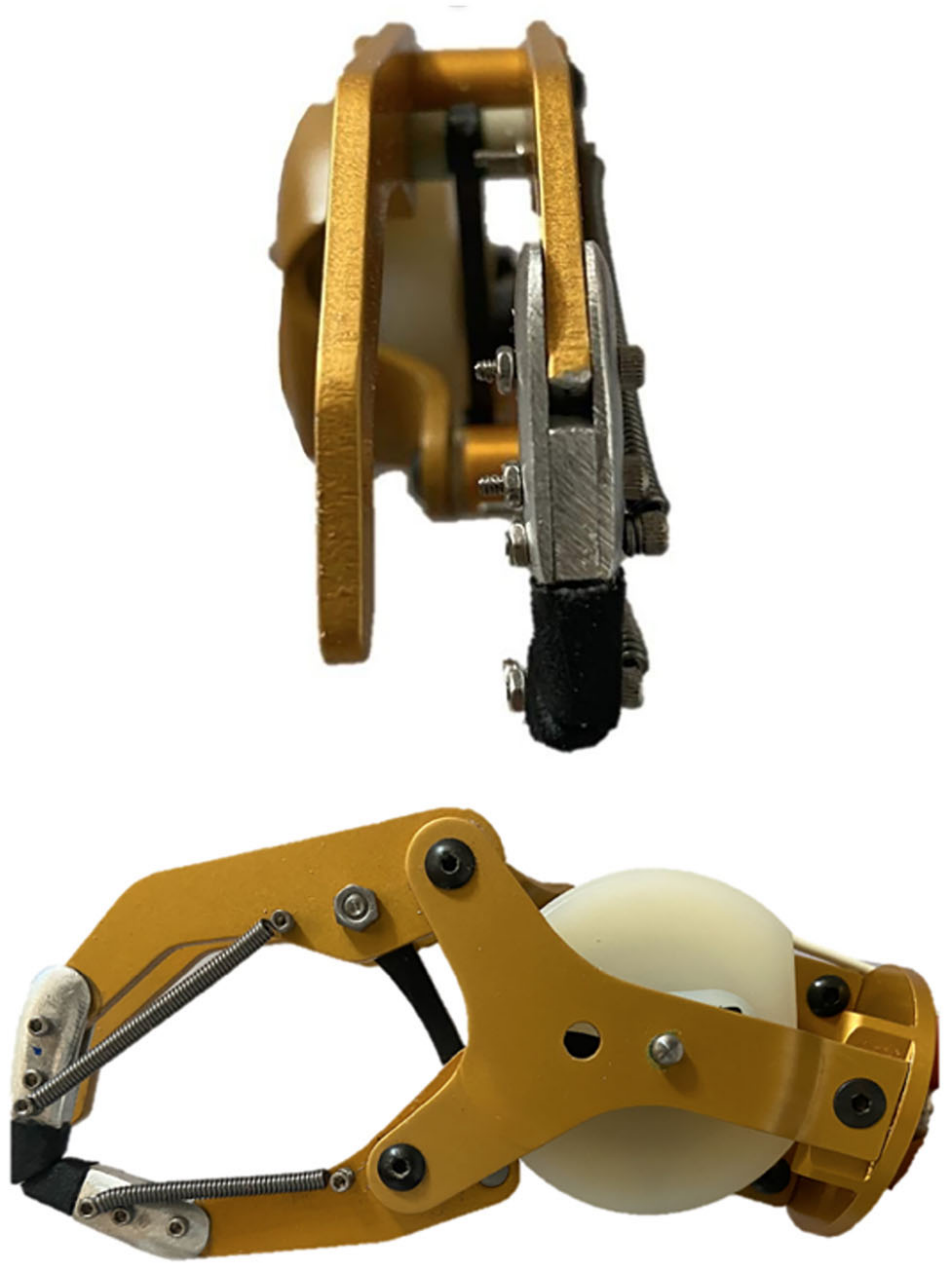
Image of a frontal and sagittal view of IPJ-Hand.

The design of the IPJ-Hand is simple and perfectly suited for prosthetists and prosthetic technicians living in austere settings. The hand is a technical modification to an existing voluntary opening (VO) prosthetic hand, the Otto Bock 8K23 (Otto Bock, Duderstadt, Germany). Importantly, any voluntary opening hand may suffice, however, when modifying the hand it is important to note that modification may void the warranty and original functionality. This hand was chosen as it is widely available in RLE, with other manufacturers offering similar hands at a range of prices. We estimated materials and modifications to these hands to be roughly $20. Fabrication begins by cutting off approximately 1.27 cm of the index and thumb fingers (Figure 1(A)). These pieces are not to be discarded as they will now serve as a template for creating six aluminum alloy extension pieces (Figure 1(B)). The exact contours of these extensions should follow that of the hands remaining fingers. This step is part art and requires only that the dimensions of each piece be a length of approximately 21 mm, a width of 10 mm and a height of 1.5 mm. Next, a series of 0.3 mm holes must be punched into the extension bars which serve as a means for attachment back onto the hand. Three bolts along with nuts are required so that the extensions can be pieced together back onto the hand. Technicians may trial a variety of bolt sizes and select appropriately. The dimension of the bolt was chosen so as to permit the hook of the springs to easily catch onto the bolts (Figure 1(C)). The final step in fabrication is to attach two stainless steel helical tension springs (Figure 2).

Springs were chosen in order to provide a pinch force between 9 and 15 N, whilst still allowing for an outer glove in order to maintain cosmesis. For the index finger spring, the free length measurement is 45 mm, the length of the body is 39 mm, the inside diameter is 0.15 mm, the outside diameter is 3.0 mm and the wire diameter is 0.08 mm. The thumb spring free length is 35 mm, length of the body is 29 mm with identical wire and inside and outside diameters. These springs were first attached to the most distal bolts on each finger and thumb extension and then to the hand itself. With the springs attached to the extensions, the opposite ends of the springs must then be attached to the proximal hand, this requires drilling two additional 0.3 mm holes. Lastly, fingertips, made from ethyl vinyl acetate foam (EVA), are glued to the extensions to aide finger pinch tasks and to protect the cosmetic glove. At this point the desired pinch force can be fixed through the use of a pinch gauge, any such gauge will suffice, however, we recommend a hydraulic gauge that allows a wide range of pinch forces (B&L Pinch Gauge, B&L Engineering, Santa Ana, CA, USA). The IPJ-Hand pinch force chosen for this study was 12.3 N and is within the average pinch force of 9 to 20 N seen in the literature for prosthetic hands [20]. Once fully fabricated, this modification permits a slight amount of interphalangeal flexion in the proximal finger and thumb, which aides fine motor movements (Figure 2).

### Procedures

Each participant was evaluated by the study prosthetist for a new trans-radial prosthesis. The choice of prosthetic socket type was largely dependent upon each patient’s unique residuum, short or long and conical or cylindrical. Thus, five participants received a Northwestern-style socket, two participants received a conventional prosthesis and one participant received a Muenster-style socket. A figure of 9 suspension was provided to four participants and a figure of 8 suspension to four participants, see Table 1. Two prostheses were made per participant because the length of the hand and hook were different, however, through adjusting the forearms, prostheses lengths remained similar. Upon successful fitting, and prior to outcome measurement, each participant performed a prosthesis checkout procedure to optimize function and transmission efficiency [21]. Optimized pinch force gauging for each hand resulted in a mean force of 10 N for CH, 24 N for FH and 12.3 N for IPJ-Hand. These pinch forces were determined from pilot testing which allowed users to trial a range of pinch forces which we later selected based on user feedback.

### Outcome measurements

**Figure 3.**
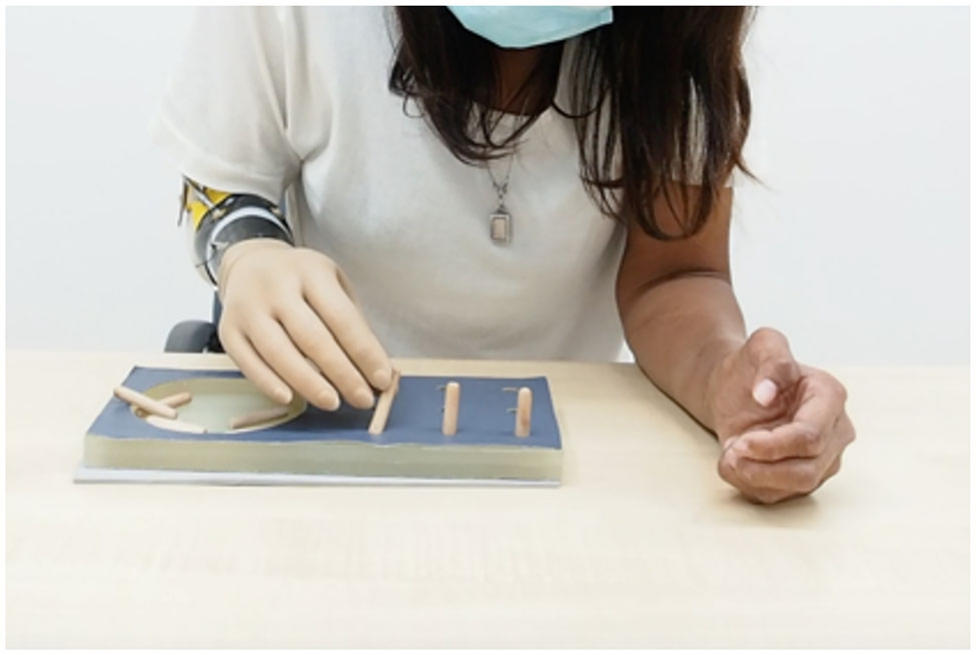
Image of a participant performing the Nine-Hole Peg Test with IPJ-Hand.

**Figure 4.**
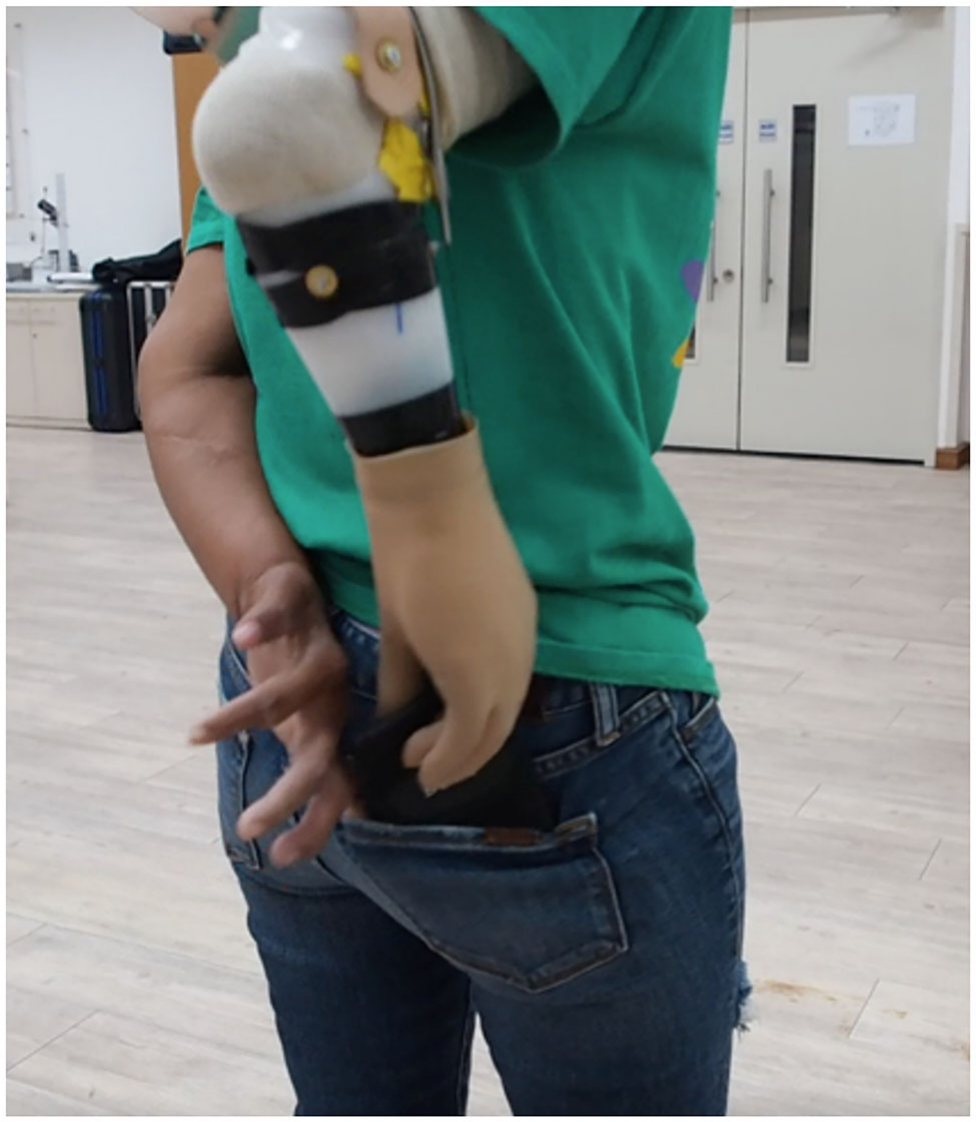
Image of a participant performing task four of the Brief Activity Measure for Upper Limb Amputees (BAM-ULA).

Each user was fit in a randomized order with either the IPJ-Hand, Conventional Hand (CH) VO Otto Bock 8K23 or Otto Bock Functional Hook (FH) VO Otto Bock 10A60. Participants then performed a pinch force test *via* the pinch force gauge and battery of outcome measurements. The same prosthetic outer glove was applied to each hand, blinding the participants to the hand type. Both performance-based and patient-reported outcome measures were administered. Outcome measurements were selected because they challenged the user to perform both large and fine motor skills and allow for subjective feedback. Practice time was provided for all performance-based outcome measures with sufficient time for participants to accommodate device and outcome measures. Once participants felt accommodated, a total of three trials were performed for outcome measures. The Nine-Hole Peg Test (NHPT) required the participant to sit in a chair at a table, pick up and place nine wooden pegs into a series of holes, then remove pegs and return to a dish. Each participant was provided ninety seconds to complete this process, and the duration of time to complete this task was recorded. Figure 3 illustrates participants placing wooden dowels into a tray during testing [22]. The Brief Activity Measure for Upper Limb Amputees (BAM-ULA) required the participant to complete ten common activities of daily living, see Figure 4 demonstration of removing of wallet task of BAM-ULA [22]. A score of 0 (inability to complete the task) or 1 (completion of the task) was given for each of the ten tasks with scores being summed. Finally, the Quebec User Evaluation of Satisfaction with Assistive Technology questionnaire (QUEST) was administered to participants. The survey asks the participant to consider the weight of the device, safety and security, durability, ease of use and effectiveness [23]. From these outcome measurement data, we performed a descriptive analysis to provide descriptive data of participant outcome measurement performances.

## Results

**Figure 5.**
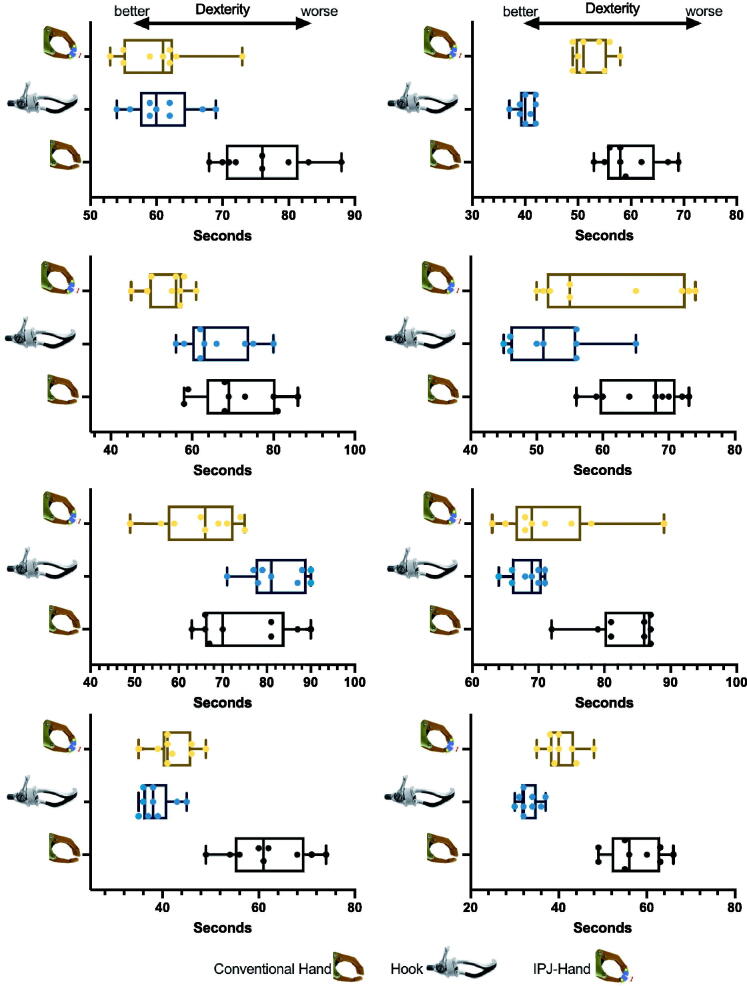
Box and whisker plots for Nine-Hole Peg Test (NHPT) in eight participants wearing prosthetic hands. Note: *: P < 0.05: the result of Paired Wilcoxon signed-rank test with Bonferroni correction after a Friedman’s χ2 r-test; IPJ-Hand: interphalangeal joint hand. Center lines show median and interquartile ranges. A longer duration indicates worse dexterity and a shorter duration better dexterity.

**Figure 6.**
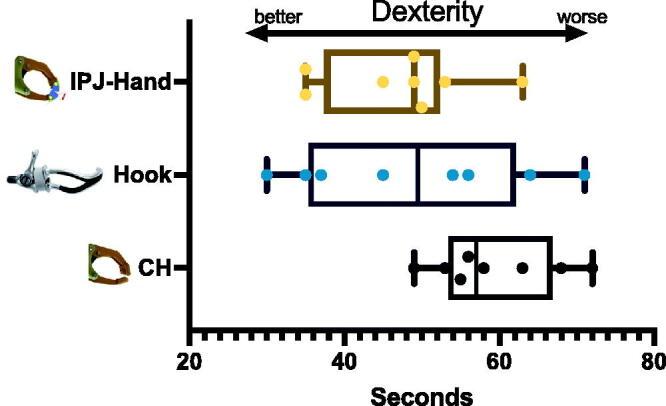
Box and whisker plots for Nine-Hole Peg Test (NHPT) in three prosthetic hands. Note: IPJ-Hand: interphalangeal joint hand; CH: Conventional hand. Center lines show median and interquartile ranges. A longer duration indicates worse dexterity and a shorter duration better dexterity.

**Figure 7.**
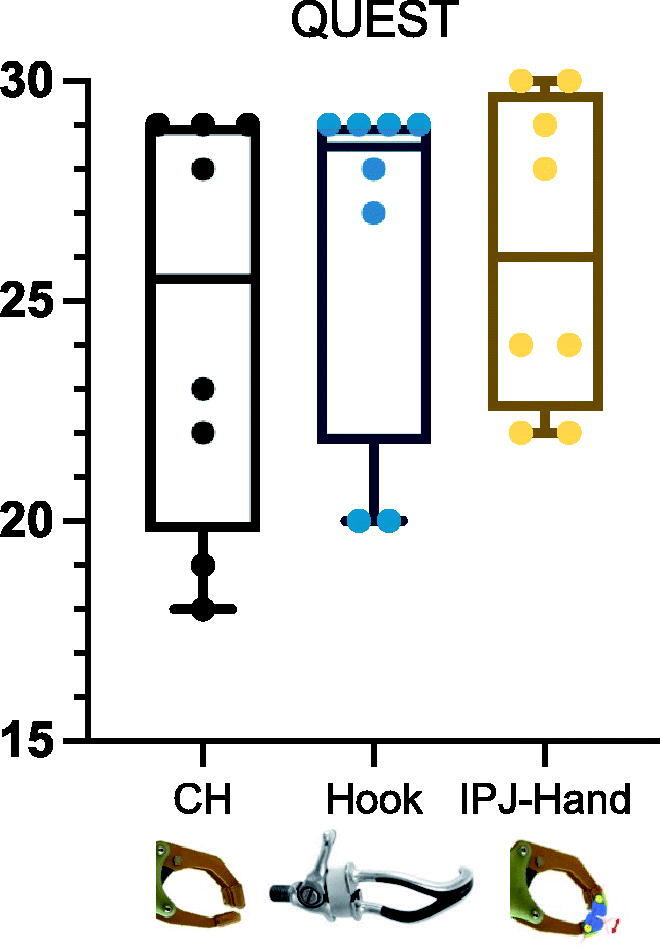
Box and whisker plots for QUEST in three prosthetic hands. Note: QUEST: Quebec User Evaluation of Satisfaction with Assistive Technology; IPJ-Hand: interphalangeal joint hand; CH: Conventional hand. Center lines show median and interquartile ranges. longer duration indicates worse dexterity and shorter duration better dexterity.

Due to the small number of subjects and skewed data distributions, the whole data was reported at the median and Interquartile range (IQR). Nine Hole Peg Test (NHPT) scores across each participant varied with large differences observed between CH and IPJ-Hand (Figure 5). Differences between hands were seen in NHPT with CH 57(13.3), FH 49.5(26.5), IPJ-Hand 49(14.8) seconds respectively (Figure 6). In the BAM-ULA, no large differences were observed across each hand with the conventional hand scores being 10(1.5), FH 10(0.7) and IPJ-Hand 10(1.7). Lastly, QUEST scores were as follows, FH 28.5(7.2) with the highest score, IPJ-Hand 26(6.8) and lastly CH 25.5(9.2) (Figure 7).

## Discussion

The objective of this study was to enhance upper limb amputee dexterity whilst maintaining cosmesis through the development of an interphalangeal prosthetic joint. Pinch forces for each of the three hands were within normal ranges for prosthetic hands [20]. Individual participant results varied, with four participants performing better on the NHPT when using the IPJ-Hand when compared to the CH and FH. Although, comparisons for each type of hand across all trials of the NHPT indicated no differences between IPJ-Hand and CH. No differences between hands on the BAM-ULA were seen, and although no large differences in QUEST were observed, users performed best using the IPJ-Hand.

Upper limb prosthesis hand development has seen marked improvements over the last decade. This is especially apparent with the advent of additive manufacturing technologies which have ushered in a new method of creating prosthetics [24], and the possible use of carbon fiber for lightweight durable prosthetics [25]. Moreover, additive manufacturing is cost-effective, rapid and can greatly enhance the research development and innovation activity of prosthetics. A plethora of materials can be processed with 3D printing (e.g. polymers, composites, metals, alloys), and provides freedom when complex models and structures have to be manufactured.

Creating a prosthesis with intuitive control and an aesthetic which can help the users overall body image is a goal worth striving for in prosthetic treatment [26]. Projects like the Defense Advanced Research Project Agency (DARPA) have greatly enhanced the possibilities for upper limb amputees [27]. Still, as helpful as these technologies are, for now at least, the dexterity offered by these devices remains out of reach for the majority of those with limb loss. These devices are myoelectrical controlled *via* electromyography (EMG) and although they offer great dexterity [28,29], these users tend to depend upon visual feedback of their device for hand awareness which can increase cognitive demand on function [30]. In fact, participants of our study performed better when using the FH over the CH. This may be because functional hooks do not interfere with the line of sight of an amputee as hands sometimes may [7]. However, whether or not CH users required more visual attention or cognition was not explored in our study.

In contrast to more recent myoelectrical prosthesis research, our study participants could not benefit from EMG. It may very well be that participants employed a feedforward control system through the formation of an internal model of their arm comprising of prosthesis weight, length and segment masses [31,32]. This internal model may have aided body-powered prosthesis users through direct hand-to-prosthetic harnessing proprioceptive feedback [33]. However, we did not collect these data, and future research is needed to determine whether IPJ-Hand users can leverage an internal model, by enhancing tactility through IP joint modification. Still, identifying the optimal spring attachment points for IPJ-Hand required numerous iterations, however once determined, lead to optimal pinch forces for all participants. This process is slightly trial and error, and the clinician or technician will have to contend with this during fitting.

This study has several limitations that negate generalizing findings to the broader community. Our sample size (*n* = 8) was small and recruited experienced prosthesis users. The preliminary character of this study necessitates a more robustly designed trial before we can recommend the broad use of the IPJ-Hand. A within-subjects design recruiting a larger cohort of participants would add greater statistical power to the study. After performing a post hoc analysis we determined that a sample of 14 can help achieve a more suitable power (1 − β = 0.83). Another limitation of our study was the limited duration provided to use each type of hand before outcome measurement. This is especially important with patient-reported outcome measures of satisfaction such as the QUEST which should be evaluated after a longer free-living experience. However, our participants expressed positive feedback, often requesting to trial the hand in the home during activities of daily living. Moreover, because the IPJ-Hand is covered with a hand cover, they noted increased confidence and a willingness to use the hand in public settings. Users noted that the IPJ-Hand combined with a body-powered harness offered the capability to sense their pinching force through the harness, even when closing their eyes. Still, we recommend others to evaluate the long-term use of this hand in free-living settings. Moreover, some research has observed improved prosthesis use and skill with longer accommodation time for hand training [34].

## Conclusion

The IPJ-Hand adds to the many prosthetic technical interventions a prosthetist and technician can perform to enhance user quality of life. Although the IPJ-Hand was initially intended for those in RLE, users in developed settings preferring body-powered systems but in need of additional dexterity may benefit from this hand. Scholars have surveyed persons with limb loss and asked them to list their top five design priorities for a prosthesis. A life-like appearance and enhanced dexterity were among the top requests [14]. We hope the IPJ-Hand may someday facilitate dexterity and aesthetics for upper limb prosthesis users.

## Data Availability

The data that support the findings of this study are available from the corresponding author, K.S., upon reasonable request.
